# Participant views on participating in a pragmatic randomised controlled trial: the Aboriginal and Torres Strait Islander Women’s Fitness Program

**DOI:** 10.1186/s12939-014-0077-3

**Published:** 2014-09-06

**Authors:** Karla Canuto, Robyn McDermott, Margaret Cargo

**Affiliations:** School of Population Health, University of South Australia, Adelaide, Australia; Wardliparingga Aboriginal Health Unit, South Australia Health and Medical Research Institute, Adelaide, Australia

**Keywords:** Pragmatic RCTs, Qualitative research, Aboriginal and Torres Strait Islander, Physical activity, Randomisation

## Abstract

**Introduction:**

The inequity of randomising participants to control groups in randomised controlled trials (RCTs) is often considered inappropriate, especially for research trials that include vulnerable populations such as Indigenous peoples. The Aboriginal and Torres Strait Islander Women’s Fitness Program conducted a trial that randomly assigned participants to ‘active’ and ‘waitlisted’ groups. This paper reports on participant views of the randomisation protocol.

**Methods:**

A pragmatic RCT was conducted in an urban setting to assess the effectiveness of the 12-week Aboriginal and Torres Strait Islander Women’s Fitness Program on metabolic health outcomes and waist circumference. Qualitative interviews were conducted at follow-up, one of the objectives was to explore participant perspectives on the research protocol, including participant randomisation to ‘Active’ and ‘Waitlisted’ groups.

**Results:**

A total of 49 interviews were conducted (26 Active and 23 Waitlisted participants). Two key factors influenced participant views on the protocol: 1) group assignment; and 2) how well they understood the research design, including the justification for randomisation. ‘Active’ participants were concerned about the inequity of the randomisation process but overall supported the study protocol. Although most Waitlisted participants were disappointed about having to wait 12-months for the program, some participants derived motivation from being waitlisted, whilst others lost motivation. Well-informed participants were more likely to express both support for the randomisation process and an understanding of the research benefits than participants not attending an information session prior to registration.

**Conclusions:**

Participants were more accepting of the research protocol if it was clearly explained to them, if they understood the randomisation process and felt the randomisation was justified in terms of the potential for the results to benefit other Aboriginal and Torres Strait Islander women. Our study suggests that the time and resources required to adequately explain the research protocol in research trials should not be undervalued.

**Trial registration:**

Australian New Zealand Clinical Trials Registry (ACTRN12610000224022).

## Introduction

The randomised controlled trial (RCT) is considered the highest level of evidence for evaluating intervention effectiveness [1]. Some of its design features, such as double blinding and placebos are not possible when evaluating behavioural interventions implemented in real world settings. Pragmatic RCTs are commonly used to evaluate lifestyle interventions because they utilise many strengths of an RCT, such as the random allocation of participants to intervention and control groups, which reduces the possibility of unmeasurable differences between groups. Pragmatic RCTs have less stringent inclusion criteria to better reflect the heterogeneity of participants and increase external validity [2]. This design, however, remains open to internal validity threats such as contamination across groups, compensatory rivalry and resentful demoralisation of participants assigned to the control group.

The ethics of randomising participants into a control group that has little or no intervention has been debated [3,4]. Some Indigenous health research trials have intended to conduct an RCT but revised their design based on feedback from the community or health workers that it was an inappropriate design [5,6]. Given these concerns, our RCT trial design utilised waitlisted control groups to evaluate the Aboriginal and Torres Strait Islander Women’s Fitness Program. This study reports on how participants enrolled in a pragmatic RCT felt about the research design and, in particular, the randomisation of participants into the active and waitlisted groups.

## Methods

### Study design

The pragmatic RCT design required registered participants to be randomly assigned to either an active or waitlisted group. Randomisation was conducted using PEPI, an online program for epidemiologists. Each wave of participants was randomly split into two groups of equal size. The waitlist control design was considered by investigators and the advisory committee to be an acceptable compromise and preferable to the traditional control group. Both groups completed health assessments and surveys at baseline (T1). The Active group participated in the 12-week fitness program shortly after their baseline assessments. Assessments for both groups were taken immediately after the 12-week program (T2), and then approximately 3-months (T3) and 9-months following program completion (T4). T4 was approximately 12 months from T1 and became the waitlisted group’s pre-program assessment. The study design is described in more detail elsewhere [7].

### Recruitment

The study invited Aboriginal and/or Torres Strait Islander women aged 18–64 years, living in the Adelaide metropolitan area to enrol in the trial. Participants were excluded if they were pregnant or had a chronic medical condition and could not obtain medical clearance. They were required to have a waist circumference greater than 80 centimetres, which was assessed at baseline (T1) prior to randomisation. The waitlisted period was a compromise between the ability to assess long-term outcomes, which ideally would have been 12 months post program (15 months from baseline), and the pragmatic issues of avoiding the 12-week program falling during mid-year school holidays or the Christmas period.

### Participant information

**Figure 1:**
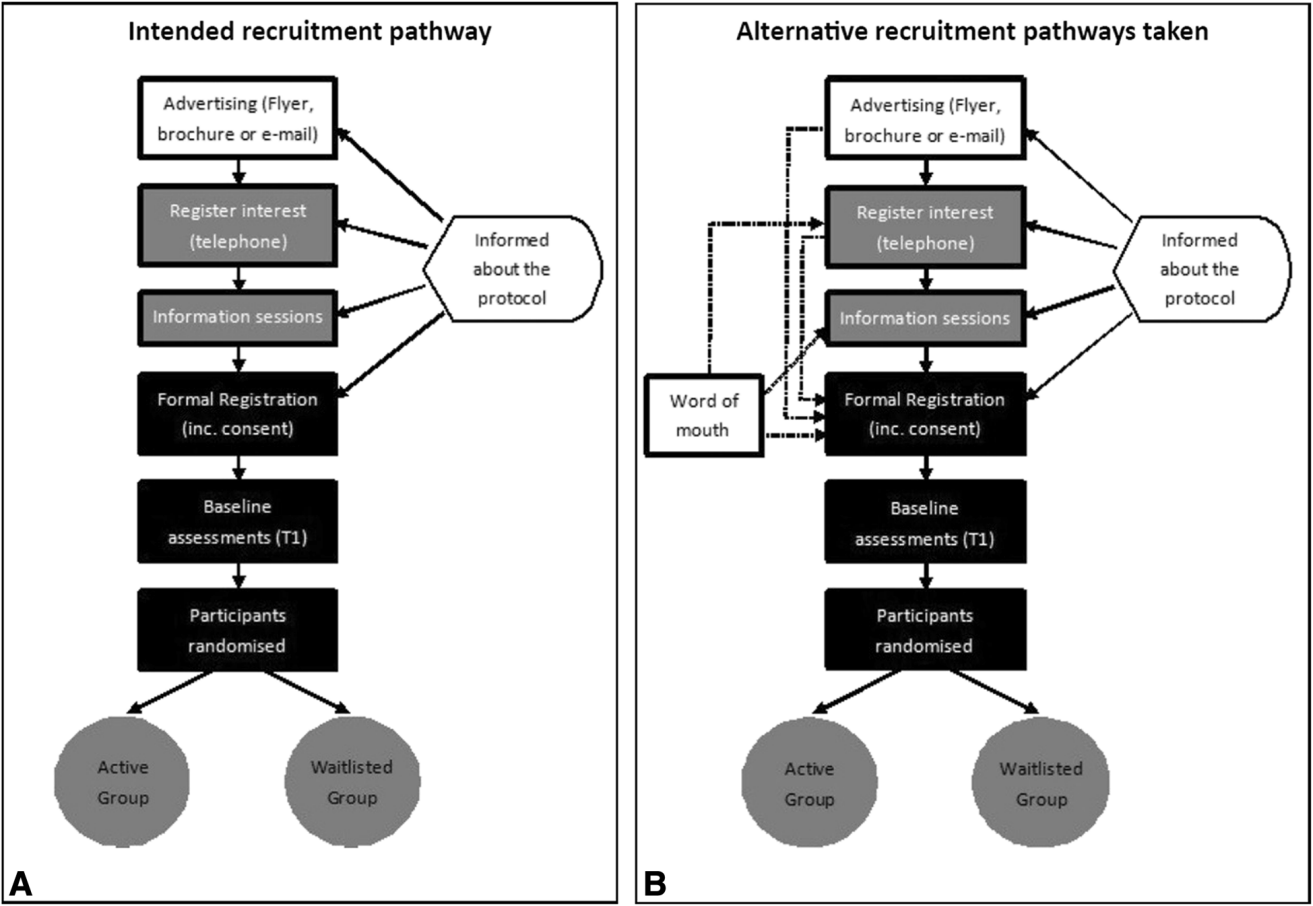
**The intended recruitment pathway of participants into the trial (A) and the many alternative recruitment pathways taken (B).**

The recruitment process was designed so that participants would receive information about the research protocol in written and verbal forms, multiple times. This, however, did not always occur. Figure 1A depicts the intended pathway of recruiting participants through to randomising participants into Active and Waitlisted groups. Advertising materials encouraged interested individuals to telephone or e-mail researchers for additional information. During initial contact researchers explained the study purpose and research protocol. An emphasis was placed on explaining the eligibility criteria and randomisation of participants. In addition, several information sessions were held during each recruitment phase. These one-hour group sessions provided potential participants an opportunity to hear from the researchers and ask questions. The information sheet and consent forms used for formal registration provided another opportunity for researchers to discuss the trial with each participant. The participant information sheet included a comprehensive plain language explanation of the research protocol. Figure 1B illustrates the alternative pathways (shown as the broken arrows) in which some participants enrolled in the program. This was predominately due to potential participants finding out about the program through word-of-mouth or if they were unable to attend the formal registration or an information session. For example, of the 37 participants who formally registered during the second recruitment wave only 20 (or 54%) attended the information session.

The information sessions were critical. They provided comprehensive information on the program and were facilitated by the PhD candidate (KC), a Torres Strait Islander woman. Several information sessions were held for each recruitment wave, in various locations including the outer suburbs. Although the sessions were quite informal the project was systematically explained including; why the trial was important, what it hoped to achieve and what participants could expect. There was a specific emphasis on the randomisation of participants; why, how and the implications. Open discussion was encouraged and participants were able to voice their concerns and have their questions answered. The information session was also used to gauge participant preferences and requirements for the group sessions including the most convenient times and days for sessions and if crèche or transport was required. Tea, coffee and fruit were provided. Children were welcome to these sessions and transport was organised if required.

The Waitlisted group received a pedometer and a 12-week exercise diary shortly after randomisation. Participants were encouraged to aim for 10 000 steps per day and record their steps. They received a monthly newsletter that included physical activity and nutrition tips including one healthy, low budget, easy to prepare recipe. Health workshops were held for the Waitlisted groups during their 12-month waiting period. Waitlisted participants were not discouraged from joining any exercise or weight-loss programs during the 12 month wait period. The Waitlisted group acted as controls and were invited to four anthropometric and metabolic health assessments in-line with their ‘Active’ group counterparts [7].

### Data collection

Information on participant age, body mass index (BMI) and employment status was obtained from the baseline assessments [7], and exercise class attendance was recorded at each session by project staff [8]. Additional assessments were taken that are not described in this manuscript.

Participants from both groups who had completed their post-program (T2) anthropometric assessments (36 Actives and 35 Waitlisted) were invited for an interview. Interviews were conducted one-on-one by an experienced female Aboriginal interviewer over a period of a few weeks for each wave of participants. Semi-structured interview guides were used; one for the active participants and one for the waitlisted participants. The venue for interviews was negotiated with each participant. The interviewer was flexible and conducted interviews after hours, at the University, participants’ workplaces or homes - where and when the participant preferred. Some interviews were conducted over the telephone if a face-to-face session could not be arranged.

The study was informed by Chen’s program planning framework, a provider based framework for understanding how factors internal and external to the program environment influence implementation and program outcomes [9]. Participant-level factors are a key dimension of this framework. The interviews probed into participant perceptions of the program including the research protocol and their experiences with randomisation. The interview guides started with questions on participants’ experiences with joining the program such as, ‘How did you hear about the Women’s Fitness Program?’, ‘What influenced you to sign up?’ and ‘What were/are you hoping to get out of the program?’ Participants in the Active groups were asked additional questions about the 12-week group sessions, probing around their experience and any barriers or enablers to their engagement. Participants raised issues concerning the research protocol and the randomisation at different points in the interview. The issue of randomisation was probed for by the interviewer if it was not raised by the end of the interview.

Interviews were digitally recorded and transcribed by a professional service. The interviewer completed a contact summary sheet upon completion of each interview. The contact sheet captured information such as the quality of responses, main issues or themes as well as body language and general comments [10]. The contact summary sheets were used to contextualise the interview, for instance, interpreting the interview in relation to the willingness of the participant to share their story. Additional information on the interview protocol is published elsewhere [8].

### Analysis

Stata version 11 was used to analyse and report descriptive data on participants [11]. Qualitative interviews were digitally-recorded, professionally transcribed and analysed using NVivo Version 9 [12]. The lead author completed the initial identification of ‘meaning units’ related to the study protocol and participant randomisation [13]. Interviews were analysed chronologically, by group, with the analyst alternating between the active and waitlisted participants until all interviews were analysed. A conceptually ordered display was used to explore and describe the reactions of participants assigned to the Active or Waitlisted groups [10]. Segments of text from the interviews of relevance to the research question were assigned codes [10]. Codes were compared and contrasted and assigned to themes. The framework was iteratively refined from the constant comparison of codes between the two groups. Interpretations of the data were additionally refined by checking participant attendance to the information and formal registration sessions highlighted in Figure 1. Two of the authors independently coded the interviews and achieved consensus on the codes through discussion. The study protocol was approved by the Human Research Ethic Committees of the University of South Australia (reference number P006/09) and the Aboriginal Health Council of South Australia (reference number 04-09-298).

## Results

**Figure 2:**
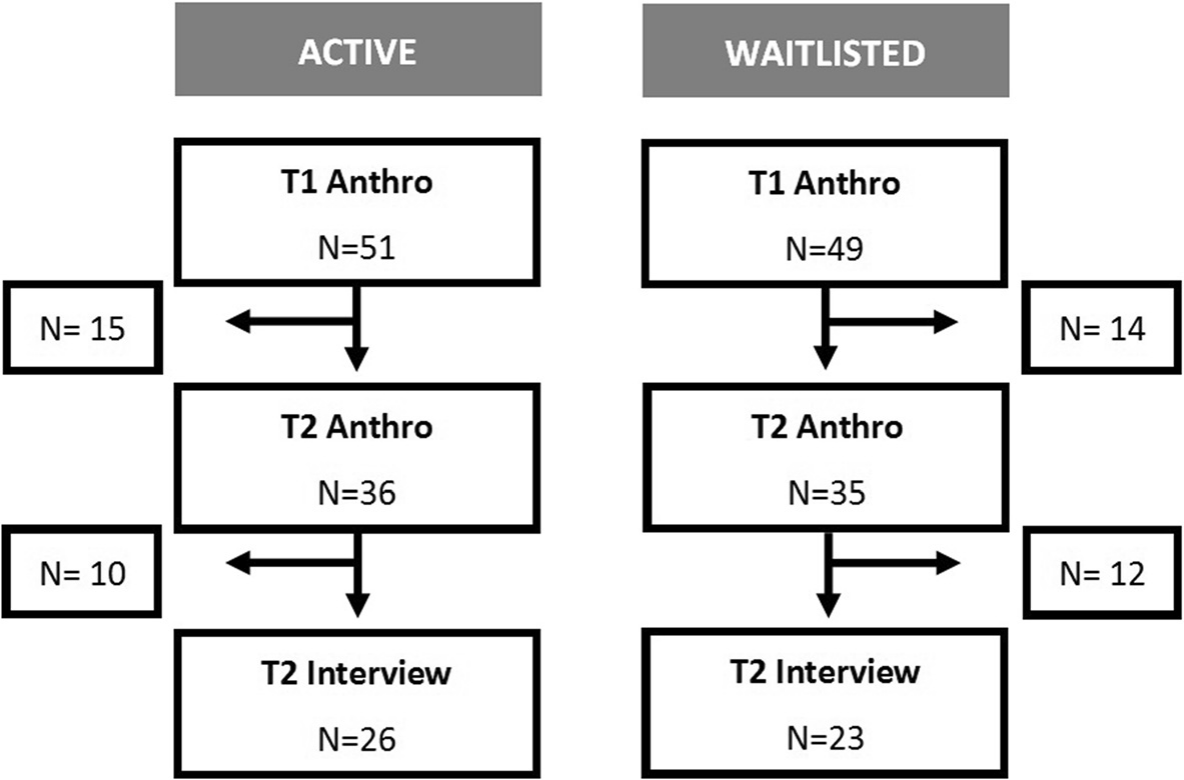
**Flow chart of participant engagement in the program from baseline anthropometric assessments (T1), through to T2 interviews.** Abbreviations: Anthro = anthropometric assessments, which included height, weight, waist and hip circumference.

Nearly 70% of participants invited to a T2 interview, completed one. Of the 49 participants who completed an interview, 47% were from the Waitlisted group. Overall, 100 participants formally registered and completed their baseline (T1) assessment. Seventy-one participants (36 Actives and 35 Waitlisted) completed post-program (T2) health assessments and were subsequently invited for a T2 interview. Of these, 49 completed a T2 interview, 47% of whom were from the Waitlisted group. A flow chart of the participants from the Active and Waitlisted group is shown in Figure 2, which depicts the sample interviewed. Further information regarding participants lost to follow-up is published elsewhere [14].

Table 1 presents descriptive data for participants who were interviewed or not interviewed, by group. Participants who did not attend their T2 anthropometric assessments (immediately after the 12-week program), were not invited for a T2 interview (15 Actives and 14 Waitlisted). Of the participants who were invited, but did not have a T2 interview, 10 were from the active group and 12 were from the waitlisted group. Participants who completed a T2 interview were similar in age and BMI compared to those who did not complete an interview, even across groups. Within each group participants who completed an interview were more likely to be employed and to have children living in the household. Additionally, Active group participants who were interviewed attended more group exercise classes than those not interviewed.

**Table 1:** **Baseline characteristics and mean exercise class attendance of interviewees and non-interviewees, by group**

	**Active N = 51**		**Waitlisted N = 49**	
	**T2 Interview**	Not Interviewed*	**T2 Interview**	Not interviewed*
	**N = 26**	**N = 25**	**N = 23**	**N = 26**
**Age at T1** Mean (95% CI)	40.7 (36.2 – 45.2)	42.7 (35.5 – 49.9)	41.7 (37.5 – 46.0)	42.1 (36.6 – 47.6)
**BMI at T1** Mean (95% CI)	35.2 (31.7 - 38.6)	36.2 (31.7 - 40.6)	35.5 (31.7 - 39.2)	33.8 (30.1 - 37.6)
**% Employed** ^**#**^	73.0%	32.0%	87.0%	34.6%
**% Kids at home**†	46.2%	32.0%	47.8%	30.8%
**Classes Attended** Mean (95% CI)	13.3 (10.9 – 15.7)	8.7 (4.0 – 13.4)	N/A	N/A

Abbreviations: T1 = baseline assessment time point, T2 = second assessment time point (post intervention), BMI = body mass index, N/A = not applicable.

*The participants ‘not interviewed’ includes all participants who attended their baseline anthropometric assessment but were not interviewed at T2. Please note this includes participants who withdrew from the trial or were lost to follow-up.

^#^“Employed” includes part-time, casual and full-time employment

†“Kids at home” includes any number of children, under 18 years of age, residing in the household.

The average interview length was 41 minutes for the Active participants and 17 minutes for the Waitlisted group. This was reflective of the fact that Active participants were asked several additional questions about the 12-week exercise program, which of course were not applicable to the waitlisted group at the time. The majority of interviews were conducted in-person in a quiet environment. The quality of the interviews was judged to be quite high, however there were a few difficult telephone interviews; one with a poor telephone connection and two with children playing loudly in the background.

**Figure 3:**
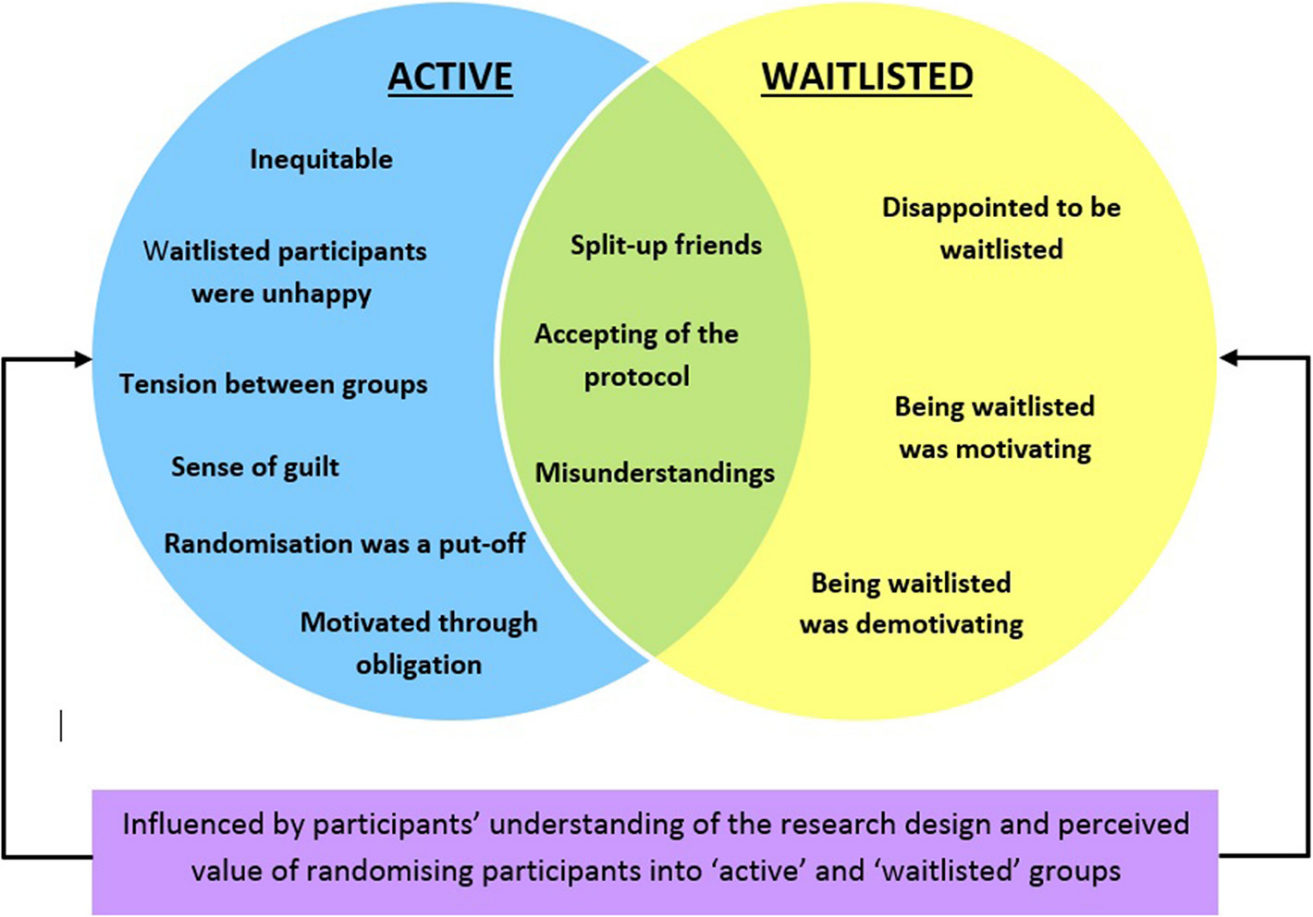
**Venn diagram of the most common issues expressed about the randomisation protocol by the participants.** Views of Active participants on the left and Waitlisted participants on the right. The overlapping section represents views expressed by both the Active and Waitlisted groups.

Two key factors were identified as influencing how participants felt about participant randomisation: 1) which group they were randomised into, and 2) their level of understanding about the protocol and why the study design was chosen. The Venn diagram (Figure 3) depicts the most common issues expressed during the interviews. The left-hand circle represents issues expressed by the Active group and the right-hand circle the Waitlisted group. The overlap between the circles represents themes common to both groups. The themes bolded in the results section correspond with the Venn diagram.

### Active group

Participants in the Active group expressed significant concern for their Waitlisted counterparts. Specifically, they were concerned about the randomisation being inequitable and reported that the Waitlisted women weren’t happy about their allocation and concern that participant randomisation may have put people off from joining the program or led them to drop out of the program. There was also a sense of obligation to make the most of the program since they were *lucky* enough to be in the active group while others had to wait. There were a number of active group members who felt well informed about the research trial and were supportive of the design whilst others understood the rationale for the protocol yet remained uncomfortable with the randomisation process.

Some Active participants felt that randomisation was ***inequitable*** and questioned whether it was ‘fair’ to randomise motivated women into a group that had to wait 12 months for the program.

*“Yeah one of the things that we were really concerned about at the beginning were the woman who actually applied and then didn't get in that was a really, really, sad, sad thing to hear and I understand that it's a research program and all this sort of stuff but I thought it would have been good for them as well to have the opportunity to participate in a different way”* (Active)

In addition, a couple of Active participants reported that some ***Waitlisted participants were unhappy***; that they were ‘angry’ or ‘upset’ about being allocated to the Waitlist group.

*“…I mean a lot of them people I'd heard we're really angry when they didn't get in and didn't want to be a part of the program at all after that and I think that’s unfair.” (Active)*

*“…those who didn’t get in were really quite upset about not getting in and I don’t know how you get around that…” (Active)*

In contrast, only one Waitlisted participant described being ‘annoyed’ when she was notified that she was in the Waitlisted group.

*“…just like annoyed I suppose. I don’t know.”* (Waitlisted)

Active participants also felt that the ***randomisation was a put-off.*** They thought that the research design may have deterred people from joining in the trial or led participants to drop out, especially if they were allocated to the Waitlisted group.

*“I think we've lost some women who won't now participate and who may have tainted other people opinions about coming along and participating”* (Active)

*“Well, when I put my name down for this program, another work colleague of mine, you know, we both signed up together, but she didn’t get picked in this round here, she got the other one where she wasn’t doing the exercise classes (Waitlisted group), and then, I mean she’s gotten real slack now, and she sort of can’t wait until the next exercise class starts, and she’s pulled out.”* (Active)

The Active group also thought that randomisation had caused ***tension between groups***.

*“… a lot of us, we’re friends and that, we all applied together and then some got in and some didn’t … that causes tension because everyone knows everyone…”* (Active)

Whilst others expressed ***a sense of guilt*** because they were in the Active group.

*“I saw the disappointment in my co-workers when they hadn’t gotten chosen and the lack of motivation as well … I felt guilty in a way.”* (Active)

No such tensions were reported in the interviews with those in the waitlisted group.

The randomisation process gave some Active participants additional motivation. Some perceived themselves as lucky and were therefore ***motivated through obligation*** and felt they had to make the most of the opportunity, especially since others had ‘missed out’.

*“… they had to recruit the groups and you know, a lot of us, we’re friends and that. We all applied together and then some got in and some didn’t, but in a way for me because I got in, then I felt that I had a commitment to the two people that I applied with that didn’t get in …”* (Active)

Allocation to the Active group seemed to instil a sense of commitment in some of the women.

#### The Waitlisted group

Participants in the waitlisted group were more likely to describe how randomisation into the Waitlisted group made them feel, to which the overwhelming response was ‘disappointed’. Some however, found being waitlisted as motivating as it gave them an opportunity to prepare and make the most of the program after their waitlisted period. Understandably, however, some participants found being randomised into the waitlist group completely demotivating as they had been ‘ready’ to start when they enrolled. Some waitlisted participants expressed an acceptance of the randomisation protocol regardless of how it made them feel.

Almost all of the waitlisted group admitted that they were ***disappointed to be waitlisted.***

*“I guess initially I was disappointed, obviously because you build yourself up to go, oh this is going to be so awesome, I'm going to get fit, I'm going to meet all these girls and do all this stuff and it was so exciting. And then you sort of get told that you're on a Waitlist, that you have to wait a year and it's sort of like, well am I still going to be motivated to go in a year?”* (Waitlisted)

*“I was sort of disappointed but it wasn’t, it wasn’t detrimental, I still know that I’ll be doing the group and I’ll be doing it next year, it's just that I have to have a bit of patience that’s all.” (Waitlisted)*

Regardless of their disappointment, some participants actually reported that ***being waitlisted was motivating*** for them.

*“There was an initial bit of disappointment when I had to wait for 12 months to be in the second group, but in saying that I think from a psychological point of view it’s been good in preparing myself. It still feels like I had a responsibility in doing the diary; that kind of motivated me a little bit in having to still report and be accountable but even if you look in my diary you could reflect my enthusiasm for doing it”* (Waitlisted)

Other Waitlisted participants, however, felt that ***being waitlisted was demotivating***.

*“I think probably took a bit of the motivation away, because I was geared up to go and do the exercise for 12 weeks with a group, and then when I got put in the other group, I think I probably, yeah, lost a bit of motivation and just like oh well, whatever.”* (Waitlisted)

*“…(I) was ready and raring to go, whereas having to wait it's kind of like, oh okay, my motivation has dropped a little bit.” (Waitlisted)*

#### Perspectives common to both groups

Themes common to the Active and Waitlisted participants included disappointment that the randomisation process split up friends who had registered together and a level of misunderstanding around the randomisation process.

Participants often registered for the program with friends and colleagues. The randomisation process often ***split-up friends*** who registered together and this was a major concern for both groups.

*“I suppose the only thing is, I did want to be able to train with some people I knew and I suppose some of us girls said “Oh we’ll join up together” and then found out some people would be accepted and some people wouldn’t and I think some people found that really hard.”* (Active)

*“I would have preferred to be going in … because (Active participant) and me work together and she did it, so it was a bit of a bummer because it would have been nice to do it together, but it’s just how it happens.”* (Waitlisted)

Despite the concerns about splitting up friends and colleagues most participants felt well informed and were supportive of the research, including the randomisation protocol, and overall were ***accepting of the protocol.***

*“… for me anyway it was all explained. It was fairly straightforward and it was good because we were given plenty of opportunity to come and listen to the girls explain everything, the whole process… I thought it was excellent and I think that that’s needed so people have a better understanding.”* (Active)

Even if they were allocated to the waitlisted group and were disappointed by their allocation if they understood the importance of the randomisation they were more likely to be accepting of it.

*“I was disappointed and like I said at the time I totally understand why it was done that way because you have to. You have to have comparisons to draw from otherwise you just can’t prove anything so I understand. …* (Waitlisted – well informed)

Some Active participants were well informed and accepting of the protocol but still uncomfortable with randomising women into a Waitlisted group.

*“I didn’t really like the randomisation idea … but I understand the randomisation process that it’s necessary.”* (Active – well informed)

Unfortunately a small number of participants felt that they were not adequately informed or had ***misunderstandings*** related to the allocation of groups and felt unhappy about the protocol, many of these participants had not attended an information session during the recruitment phase of the program. Of the five participants who misunderstood the randomisation process or seemed confused about the protocol, only one had attended a participant information session.

*“I got feedback about that people were really unhappy about. People thought that there was some people who got into the program who were friends of (the researcher) rather than having to be there because they – they were people who were most in need. Now whether that’s true or not, I don’t know. These people that didn’t get in were saying that.”* (Active –who did not attend an information session)

*“I didn’t know it at that time but then you get the form to say that you start off on this one (Waitlisted group) …– I didn’t know that was how the program worked. I just thought everybody just did that fitness and then somewhere you did education but yeah it was quite different to what I thought”* (Waitlisted - who did not attend an information session)

*“I was a little bit upset… but I rang up last so you have to understand that like first in first served”* (Waitlisted – who did not attend an information session).

It would seem that attending an information session was vital for clarifying the randomisation process.

## Discussion

The interviews conveyed a range of concerns and reactions from participant’s involvement in the pragmatic RCT with waitlisted groups. These views appeared to be influenced by the group to which they were assigned and their understanding of the research protocol. Figure 1A depicted the intended recruitment process designed for participants to be fully informed of the protocol prior to enrolment by using different mediums (written and verbal) and presenting the information on multiple occasions. Some participants, however, enrolled in the project through the alternative pathways illustrated in Figure 1B and may have heard about it through the grapevine and consequently arrived at formal registration sessions without any prior contact with research staff. Participants who were well-informed seemed more accepting of the protocol compared to those who were less informed. The latter felt that either the fact that the program was a research trial or the process of participant randomisation was not well advertised or explained. Despite varying levels of understanding and views on the protocol there was an overall acceptance of the randomisation of participants.

Both groups expressed concern or disappointment over the randomisation separating friends and family members who enrolled together. In general, the Active group was concerned about the equity/ fairness of randomising participants. Active participants appeared concerned about the randomisation and often talked about how the Waitlisted group felt more strongly than the Waitlisted group did. It is unclear if this is a true reflection of how the Waitlisted group felt, which was verbalised to the Active participants or if it is the perception of the Active group, which may or not reflect the Waitlisted group feelings accurately. Waitlisted participants may be underplaying their emotions in the interview or perhaps the outspoken, upset Waitlisted participants declined to have an interview or had ‘dropped out’ of the program.

Waitlisted participants were disappointed but generally accepting of the protocol and satisfied that they would have the opportunity to participate in the program. Our findings are similar to a UK based health promotion singing intervention with older adults which found that participant’s main reaction to being allocated to the control group was disappointment [15].

In retrospect, the impact of the perceived lengthy wait time for those randomised to the waitlisted group (and thereby mitigating the drop-out rate in this group) could have been potentially addressed through a more active nutrition-based program, however this was considered not feasible given the resource constraints of the project and the potential for contamination. More significantly, a reduced waiting time might have reduced dropout rates in the waitlisted group, however the RCT study design required a full interval between baseline and first follow-up, in order to accurately compare both groups over time.

### Limitations

Study findings should be interpreted in the context of the limitations. The information was captured during one-on-one interviews with participants approximately 14 weeks after their baseline assessments; therefore, 1) the responses could have been more emotional if conducted immediately after allocation to the groups and 2) those who were interviewed were engaged in the trial and were, assumedly accepting of the protocol. Participants who may have been strongly opposed to the protocol were likely to have either not enrolled in the trial, withdrawn or dropped out of the trial. Obtaining information from participants who dropped out was difficult. Not only are dropouts notoriously difficult to engage but permission to follow them up was not sought in the original ethics application. When ethics was eventually granted the restrictions imposed meant that information freely provided by participants prior to the approval could not be used.

At the first follow-up assessments, immediately after the program (T2), 15 Active and 14 Waitlisted participants failed to attend their follow-up assessment. The ‘cost’ of conducting an RCT instead of another research design is unknown. As indicated from the interviews the study design may have led participants to drop out of the program. The potential harm caused due to the distress of being allocated to the Waitlisted group, the pressure of being in the Active group or being allocated in the opposite group to your colleagues or friends is unmeasured.

## Conclusion

Conducting research in an equitable and rigorous manner is difficult, and often one must be compromised to achieve the other. This pragmatic RCT design with waitlisted controls was considered the most acceptable compromise by the Advisory Committee and researchers, and the program’s ability to successfully recruit 100 participants is testament to this. Participants were more accepting of the research protocol if they understood the randomisation process and felt the protocol was justified in terms of the benefits to the research and consequently for other Aboriginal and Torres Strait Islander women. Our study suggests that researchers should value the investment spent to ensure the research protocol is carefully explained and understood by participants prior to their enrolment. The concerns voiced by participants, especially around the splitting up of friends and the possible biases that are introduced with a pragmatic RCT design should be considered when designing similar research trials.

The waitlist control design adds considerably to the study costs, length and logistics, compared to a simple RCT. In the initial study design phase, a multiple baseline, or stepped-wedge design was considered, which may have mitigated some of the perceived impact of randomisation, however this approach may well have had the same problems with the waiting period. The implications of this for future work could be to plan a study design with an alternative arm which includes a non-exercise intervention for example, nutrition coaching, or to plan an alternative design which is a combination of before-and-after measures in the intervention group, and compare results with a sample of age- and sex-matched controls (from a community register) who were participating in another activity not related to the trial, but who might give consent for their clinical measures to be used (anonymously) as de-facto controls.

This study highlights the importance of appropriately informing participants of the processes and justifying the protocol prior to registering for a randomised trial. From a research perspective, the framing of information in brochures and recruitment information cannot be underestimated. It may be worthwhile focus group testing the strategies used to recruit participants into RCTs and to have community advisory oversight at the early stages of the trial to get these messages right. The information sessions were essential. Similar sessions should be included in recruitment ensuring that facilitators create a culturally safe space for participants to ask questions. The interviews suggest that this could improve participant satisfaction and retention rates. Mechanisms to encourage attendance at an information session as a condition of enrolment may be required to prevent participants bypassing the planned pathway for enrolment. Additional methods may also be required to ‘catch up’ participants with all the necessary information if they cannot attend an information session. The findings from this study may also assist other researchers to have open and honest conversations during information sessions about how participants may feel, including that it is normal to feel disappointed if you are randomised to the waitlisted group and that you may feel some level of guilt if you are designated to the active group whilst your friend or family member is randomised to the waitlisted group. This additional information and reassurance could help participants feel fully informed and prepared to participate in the trial.
